# P-131. Infective Endocarditis (IE) in Persons Who Inject Drugs (PWID): Expanding Novel Treatment Methods for a Challenging Population

**DOI:** 10.1093/ofid/ofaf695.358

**Published:** 2026-01-11

**Authors:** Andrea Molin, Stephanie Spivack

**Affiliations:** Temple University Hospital, Philadelphia, Pennsylvania; Temple University Health System, Philadelphia, PA

## Abstract

**Background:**

Infectious endocarditis (IE) remains a major cause of morbidity and mortality for persons who inject drugs (PWID). In patients who require surgery, traditional valve replacement carries a high risk of prosthetic valve endocarditis if they return to substance use. Novel methods include valve replacement with a bovine pericardial cylinder, valve replacement with native tissue using a pulmonary autograft (Ross procedure), and vacuum-assisted thrombectomy to de-bulk right-sided vegetations.

**Figure:**
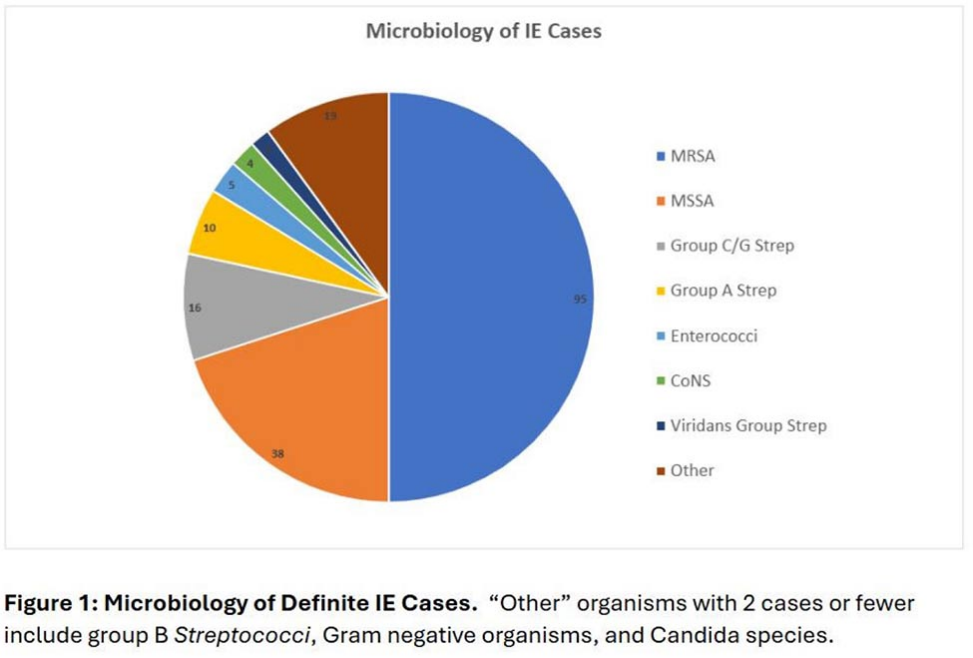

**Figure:**
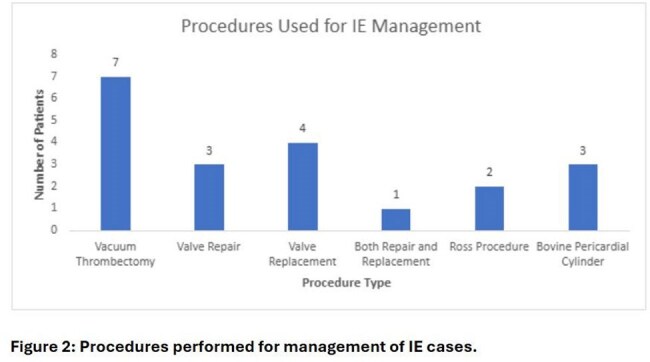

**Methods:**

We conducted an IRB-approved retrospective chart review of hospitalized PWID with positive blood cultures between March 2022 and March 2024.

**Figure:**
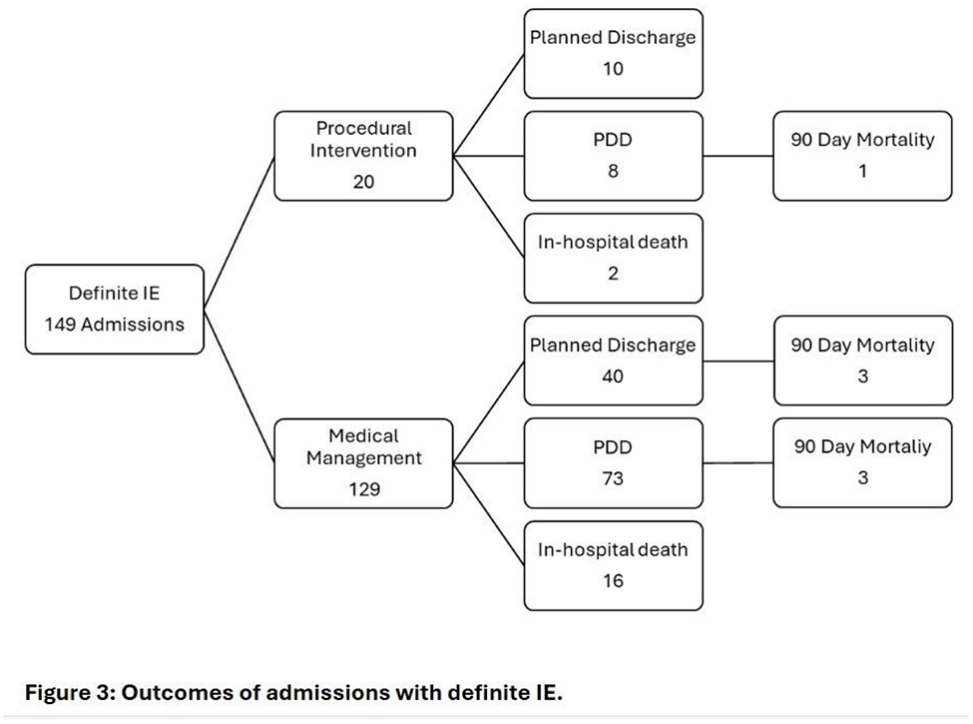

**Results:**

A total of 383 patients had 573 unique admissions with positive blood cultures. Of these, 113 patients (30%) with 149 admissions met modified Duke criteria for definite IE. Of patients with definite IE, 44% were female, the median age was 37, 68% experienced unstable housing, and 8% had prior cardiac surgery.

The most common organisms were methicillin-resistant Staphylococcus aureus (MRSA), methicillin-susceptible Staphylococcus aureus (MSSA), and group C and G Streptococci (Figure 1). The most common echocardiographic findings were tricuspid regurgitation (40%), tricuspid vegetation (37%), mitral regurgitation (16%), and mitral vegetation (15%).

Twenty patients (18%), including 2 with prior valve surgery, underwent a procedure or surgery. Procedures included vacuum thrombectomy (7), valve replacement (4), valve repair (3), valve reconstruction with bovine pericardial cylinder (3), Ross procedure (2), or both valve repair and replacement (1) (Figure 2).

More than half (54%) of discharges were patient-directed discharges (PDD), while 34% had a planned discharge. There were 18 in-hospital deaths and 7 additional deaths within 90 days. Overall, 3/20 patients (15%) who underwent a procedure died within 90 days compared to 22/113 (19%) of those who did not. One patient each had in-hospital mortality following a Ross procedure and vacuum thrombectomy; one patient with both valve replacement and repair died within 30 days.

**Conclusion:**

IE remains a major cause of hospitalization and mortality in PWID. These findings highlight the need for a variety of procedural options to meet the needs of this population.

**Disclosures:**

All Authors: No reported disclosures

